# Classifying and standardizing panfacial trauma according to anatomic categories and Facial Injury Severity Scale: a 10-year retrospective study

**DOI:** 10.1186/s12903-021-01900-w

**Published:** 2021-11-01

**Authors:** Chengzhong Lin, Jinyang Wu, Chengshuai Yang, Chuxi Zhang, Bing Xu, Yong Zhang, Shilei Zhang

**Affiliations:** 1 The 2nd Dental Center, Shanghai Ninth People’s Hospital, College of Stomatology, Shanghai Jiao Tong University School of Medicine; College of Stomatology, Shanghai Jiao Tong University; National Center for Stomatology; National Clinical Research Center for Oral Diseases, Shanghai Key Laboratory of Stomatology, Shanghai, China; 2 Department of Oral and Cranio-Maxillofacial Surgery, Shanghai Ninth People’s Hospital, College of Stomatology, Shanghai Jiao Tong University School of Medicine; College of Stomatology, Shanghai Jiao Tong University; National Center for Stomatology; National Clinical Research Center for Oral Diseases, Shanghai Key Laboratory of Stomatology, Shanghai, China

**Keywords:** Panfacial fracture (PF), Concomitant injury, Postinjury complication, Anatomic PF category, Facial injury severity scale

## Abstract

**Background:**

The purpose of this study was to identify the epidemiologic factors of panfacial fractures (PFs), and to evaluate the significance of anatomic PF categories and the Facial Injury Severity Scale (FISS) in classifying and standardizing panfacial injuries.

**Methods:**

A retrospective review of all patients treated with PFs at our institution between June 2010 and April 2021 was performed. PF was defined as a concurrent fracture in at least 3 of 4 facial subunits (frontal, upper midface, lower midface, and mandible). Data regarding patient demographics, causes of injury, location of fractures, major concomitant injuries, and postinjury complications were collected, and the FISS score was collected from each patient. Statistical analysis was performed using IBM SPSS Statistics version 22.0.

**Results:**

A total of 227 patients were enrolled. The most commonly fractured bones were the maxillary sinus wall (92.1%), mandible (82.8%), and zygomatic arch (75.3%), and the most common fracture sites in PFs were graphically presented. Four PF patterns were defined: FULM (n = 60), FUL (n = 39), ULM (n = 127), and FUM (n = 1). There was a significant association between PF patterns and sex (*p* = 0.018), the number of concomitant injuries (*p* = 0.014), and early surgical airway management (*p* = 0.003). Different PF patterns were significantly correlated with different types of concomitant injuries and complications. The FISS score showed a significant difference with PF patterns (*p* = 0.000) and sex (*p* = 0.007), and a FISS value of 11 or more is the appropriate cutoff for the prediction of multiple concomitant injuries and complications.

**Conclusions:**

Both the anatomic PF categories and FISS were significantly correlated with various concomitant injuries and complications. The combination of PF categories and FISS provided a better positive and negative prediction of concomitant injuries and complications for PF patients. Patients with FULM and FISS > 11 had an obviously higher proportion of the need for multiprofessional treatment.

**Supplementary Information:**

The online version contains supplementary material available at 10.1186/s12903-021-01900-w.

## Background

Panfacial fractures (PFs) are often the result of high-energy injuries and present remarkable challenges to both surgeons and patients. These fractures are generally defined as fractures that simultaneously involve at least three out of four subunits of the facial skeleton, that is, the frontal area, upper and lower part of midfacial area, and mandibular area [1, 2]. The various fracture patterns depend on the mechanism and degree of the externally applied forces, with the usually reported causes including traffic accidents, assaults, falls, sports and gunshot injuries. This type of trauma is often associated with emergencies, such as craniocerebral injury, thorax injury, and cervical spine injury [3–6]. Clinically, the traumatic conditions of PFs are complicated and vary between individuals.

The purpose of treating a PF patient is not only to save life but also to recover the structure, function, and aesthetics of the maxillofacial area while managing concomitant injuries effectively. The complex management of patients with multidistribution trauma requires a standardized classification for describing PF in a way that is comprehensive, measurable, validated and reproducible. Classifying PFs should be the first step toward a systemic approach for treating concomitant injuries and reducing complications [1, 7]. Traditionally, the classification of facial injuries, such as the Le Fort system [8], proposed in terms of site distribution and anatomical impairment, helps understand their impact on morbidity and complications. However, these classification standards are insufficient in their description of more complex injuries as well as injuries to the mandible, midface and upper face, which usually occur in patients with multiple concomitant injuries and complications [9]. Recently, Jang et al. [1] performed a retrospective study of 99 PF patients and classified the PFs into five categories according to the anatomical site of fractures, showing that different PF patterns were associated with different types of concomitant injuries and complications. It is suggested that this anatomic classification could be a potential tool, although these patterns convey qualitative but not quantitative information about the fracture patterns.

The use of a quantitative scale for facial fracture can be potentially beneficial, facilitating the proper treatment of concomitant injuries and complications and preventing irreversible damage. In addition, as a prognostic and predictive classification tool, it could simplify the communication among clinicians and patients about the extent of the injury [10, 11]. Although there have been several attempts to develop such a scale, there are still situations in which they are complex and lack true construct validity; thus, the viability of their use is in doubt. The Facial Injury Severity Scale (FISS) proposed by Bagheri et al. [12] has been widely used to grade the severity of facial injuries. The FISS was derived from predefined values of scores and weighting for fractures in different areas of the face. Not all fractures are weighted equally, and the sum of the individual scores is used to provide the final score. The FISS has been shown to be correlated with operative time, length of hospitalization and treatment cost of facial trauma and is considered to be the best communication tool available within multidisciplinary teams [13–16]. However, few studies have examined the predictive value of the FISS and the correlation of the FISS with concomitant injuries and complications among PFs patients.

Assessing and classifying PFs regarding concomitant injuries and complications will facilitate comprehensive treatment planning and multidisciplinary cooperation. However, such a well-developed clinical classification has yet to be reached. The purpose of the present retrospective study was to investigate the epidemiologic factors, concomitant injuries, complications, and FISS scores of PFs, and to verify the significance of anatomic PF categories and the FISS in classifying PF patients based on concomitant injuries and complications.

## Methods

A retrospective study was carried out from the data collected in hospital charts of facial trauma patients treated in the Department of Oral and Cranio-maxillofacial Surgery, Shanghai Ninth People’s Hospital, Shanghai Jiao Tong University School of Medicine between June 2010 and April 2021. The protocol of this study was reviewed and approved by the institutional review board of Shanghai Ninth People’s Hospital.

### Classification of panfacial fractures

**Fig. 1:**
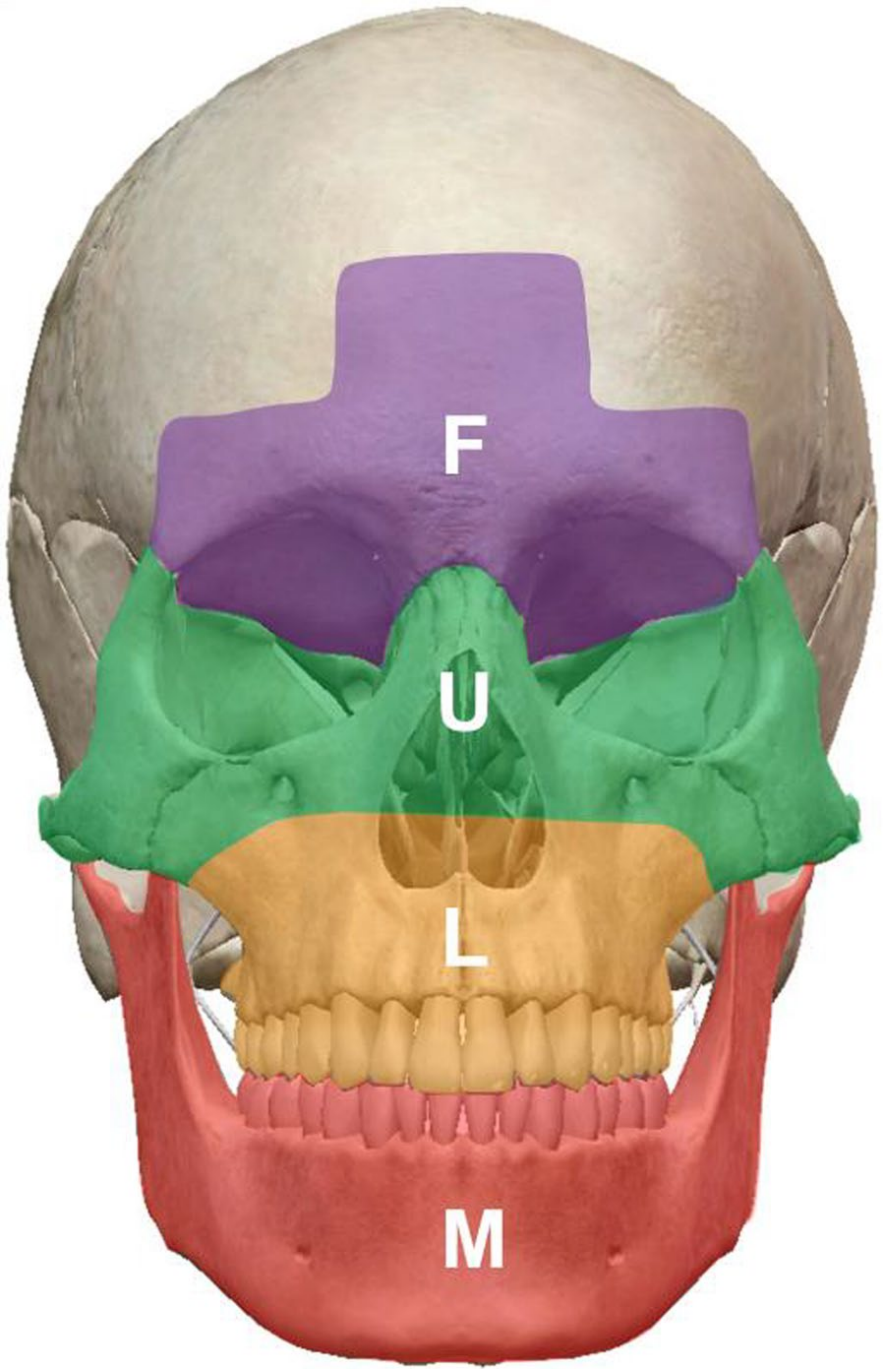
Four subunits of the facial skeleton. F: frontal unit, contains frontal bone/sinus, orbital roof. U: upper midface unit, contains lateral orbital wall, medial orbital wall, orbital floor, nasal bone, NOE area, and zygomatic arch. L: lower midface unit, contains maxillary sinus, bony palate, Le fort I. M: mandibular unit, contains all mandible. ZMC fractures, Le Fort II fractures, or Le Fort III fractures were counted as fractures of both the U and L subunits

The facial skeleton was divided into 4 subunits: frontal area, upper midface area, lower midface area, and mandible area (Fig. 1). Patients with fractures in at least 3 out of the 4 subunits were classified as PF according Erdmann et al. [2]. Hence, PFs can be divided into five categories: FULM, FUL, FUM, FLM, and ULM.

### Investigative factors

For each PF patient, the emergency department chart, all clinic notes, all relevant radiology scans and reports were reviewed. The following data were collected for each patient:

- Age/sex.
- Etiology of trauma: It was assigned as 6 categories: fall, interpersonal violence, automobile accident, bicycle/E-Bike accident, striking an object, and work accident.
- Fracture sites: The original computed tomography (CT) scan of each PF patient was reviewed to identify the exact fracture sites.
- Major concomitant injuries were classified into 5 categories: upper/lower limb injury, cervical spine injury, thorax injury, craniocerebral injury, and abdomen injury.
- Postinjury complications: Postinjury complications were analyzed for each patient.
- Facial Injury Severity Scale (FISS): The individual modified FISS score was calculated according to Erdmann et al. [2]. The point values of each fracture site in the FISS are outlined in Table 1.

**Table 1:** Duke Modified Facial Injury Severity Scale (FISS)

Fracture site	Score
Frontal sinus/bone	2
Orbital roof	1
Lateral orbital wall	1
Medial orbital wall	1
Orbital floor	1
Nasal	1
Naso-orbito-ethmoid	3
Zygomatic arch	1
Zygomatico-maxillary complex	1
Le Fort III	6
Le Fort II	4
Maxillary sinus	1
Palatal	1
Le Fort I	2
Mandibular symphyseal	2
Mandibular parasymphyseal	2
Mandibular body	2
Mandibular angle	2
Mandibular ramus	2
Mandibular sub-condylar	1
Mandibular condylar	1
Mandibular coronoid	1
Over 10 cm long facial laceration	1

The FISS is the summation of the all above diagnosed fracture points in an individual patient, unless part of a complex fractures. Unilateral Le Fort fractures are assigned half the numeric value

### Statistical analysis

Statistical analysis was performed using IBM SPSS Statistics version 22.0 (IBM, Armonk, NY, USA). The chi-square test was used to determine the significant differences between fracture patterns, FISS scores and sex, cause of injury, number of concomitant injuries, complications, and early surgical airway management. Student’s t test was used to determine the significant difference between concomitant injuries, complications and FISS score values. The cutoff value of the FISS was determined by ROC curve analysis. *p* < 0.05 was considered significantly different.

## Results

### Etiology of panfacial injuries

**Fig. 2:**
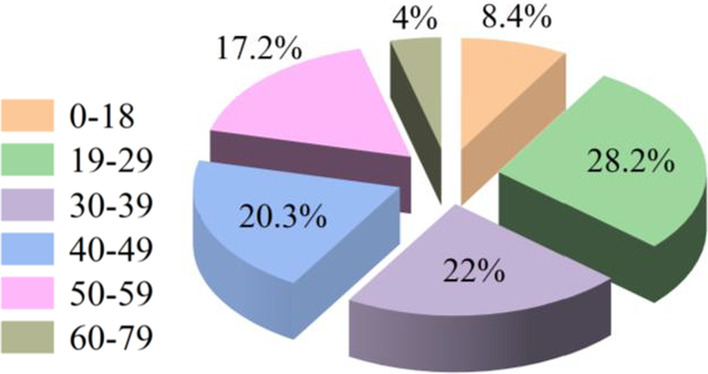
Distribution of patients by age

**Fig. 3:**
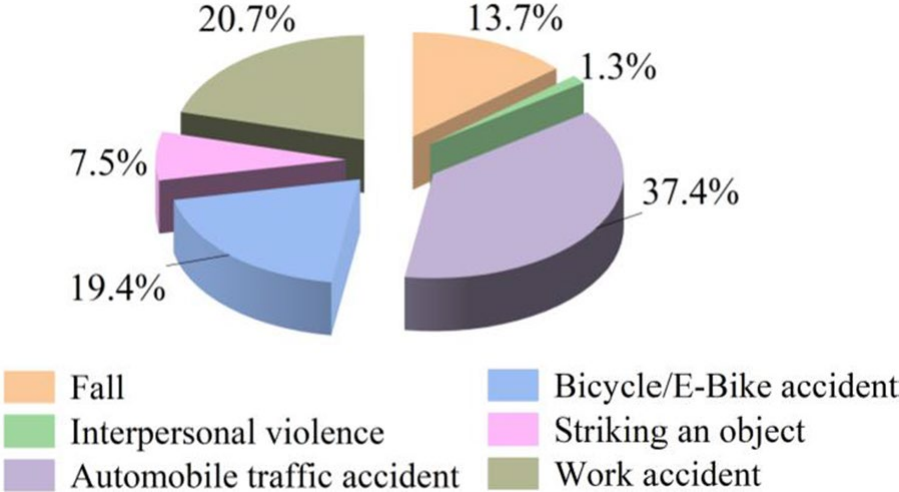
Distribution of patients depending on traumatic etiology

A total of 227 PF patients were analyzed for the inclusion criteria and enrolled in this study. Of these patients, 198 were male (87.2%), 29 were female (12.8%), and the M/F ratio = 6.8/1. The average age of the patients was 36.2 ± 14.3 years, ranging from 4 to 75 years of age, and the most affected age group was 19–29 years (28.2%) (Fig. 2; Additional file 1: Table S1). The etiology of panfacial injuries differed by age group. Automobile traffic accidents (37.4%), work accidents (20.7%), and bicycle/E-Bike accidents (19.4%) were the most common causes of panfacial injuries in all patients. In the pediatric group (0–18 years), the rates of falls (31.5%) and automobile traffic accidents (47.4%) were higher than those in the other groups (Fig. 3; Table 2).

**Table 2:** Distribution of the traumatic etiology depending on age

Age (y)	Cause of injuries						Total (%)
	Fall (%)	IPV (%)	AM acc (%)	B/E-B acc (%)	SO (%)	Work acc (%)	
0–18	6 (31.5)	0 (0)	9 (47.4)	3 (15.8)	1 (5.3)	0 (0)	19 (100)
19–29	9 (14.1)	1 (1.6)	28 (43.8)	12 (18.8)	7 (10.9)	7 (10.9)	64 (100)
30–39	9 (18.0)	0 (0)	17 (34.0)	9 (18.0)	2 (4.0)	13 (26.0)	50 (100)
40–49	5 (10.9)	1 (2.2)	16 (34.8)	9 (19.6)	4 (10.9)	11 (23.9)	46 (100)
50–59	2 (5.1)	1 (2.6)	11 (28.2)	8 (20.5)	1 (2.6)	16 (41.0)	39 (100)
60–80	0 (0)	0 (0)	4 (44.4)	3 (33.3)	2 (22.2)	0 (0)	9 (100)
Total	31 (13.7)	3 (1.3)	85 (37.4)	44 (19.4)	17 (7.5)	47 (20.7)	227 (100)

IPV, Interpersonal violence; AM acc, Automobile accident; B/E-B acc, Bicycle/E-Bike accident; SO, Striking an object

**Fig. 4:**
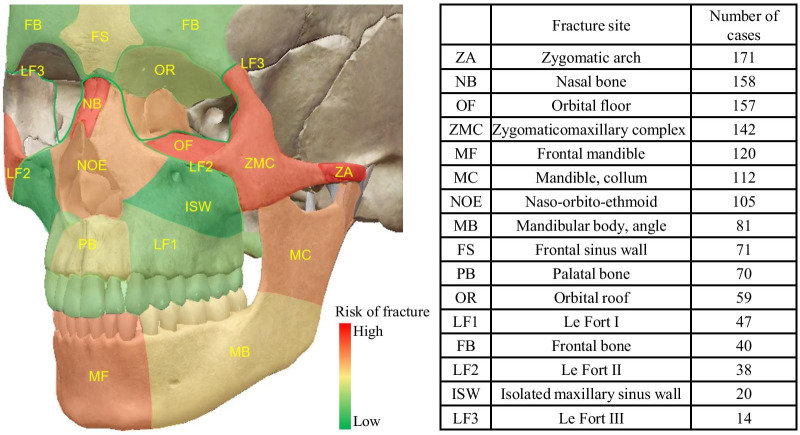
Heat map showing the areas of the facial skeleton with a higher risk of fracture

Of the 227 PF patients, the most common fracture site was the maxillary sinus wall (92.1%), followed by the mandible (82.8%), zygomatic arch (75.3%), lateral orbital wall (74.9%), and nasal bone (69.6%). Additionally, the most common sites of mandibular fractures in PF were the symphysis/parasymphysis (52.9%), body (22.0%), and condyle (20.7%). The total number of each fracture site for each mechanism of injury is presented in Table 3. In addition, the visualization of the most common fracture sites in PF cases is graphically presented in Fig. 4. The facial skull anatomic heat map revealed that these fractures usually occurred in the upper midface, such as the zygomatic arch, nasal bone, orbital floor, zygomaticomaxillary complex, naso-orbito-ethmoid complex, or in the mandibular front or collum. Le Fort III level and isolated maxillary sinus wall present a lower risk of fracture in panfacial injury patients.

**Table 3:** Fracture locations among panfacial injuries according to the cause of injury

Fracture site	Cause of injuries (A total of 227 cases)						Total (%)
	Fall (n = 31)	IPV (n = 3)	AM acc (n = 85)	B/E-B acct (n = 44)	SO (n = 17)	Work acct (n = 47)	
Frontal sinus/bone	5	1	34	20	8	16	84 (37.0)
Orbital roof	6	1	21	10	7	14	59 (26.0)
Lateral orbital wall	24	2	58	36	14	36	170 (74.9)
Medial orbital wall	15	1	52	27	15	30	140 (61.7)
Orbital floor	22	1	54	34	11	35	157 (69.2)
Nasal bone	20	2	57	31	15	33	158 (69.6)
Naso-orbito-ethmoid	10	1	38	21	10	25	105 (46.3)
Zygomatic arch	24	2	63	35	10	37	171 (75.3)
Maxillary sinus wall	30	3	76	40	16	44	209 (92.1)
Palatal bone	7	1	29	9	5	18	69 (30.4)
Le Fort I	5	0	17	15	1	9	47 (20.7)
Zygomatico-maxillary complex	18	1	52	31	9	31	142 (62.6)
Le Fort II	3	0	16	10	2	7	38 (16.7)
Le Fort III	3	0	4	3	0	4	14 (6.2)
Mandibular symphyseal/parasymphyseal	22	0	44	18	8	28	120 (52.9)
Mandibular body	5	0	26	8	3	8	50 (22.0)
Mandibular angle	2	0	7	4	3	3	19 (8.4)
Mandibular ramus	1	1	14	5	0	10	31 (13.7)
Mandibular sub-condyle	4	0	6	2	0	10	22 (9.7)
Mandibular condyle	13	1	15	3	5	10	47 (20.7)
Mandibular coronoid	1	0	4	6	0	1	12 (5.3)

IPV, Interpersonal violence; AM acc, Automobile accident; B/E-B acc, Bicycle/E-Bike accident; SO, Striking an object

### Anatomic panfacial fracture categories

Regarding the anatomic PF categories, four fracture types were defined, including FULM (n = 60), FUL (n = 39), ULM (n = 127), and FUM (n = 1). As shown in Table 4, there was no significant association between age and fracture type. The FULM pattern was mostly dominated by males (98.3%) compared with FUL (87.2%) and ULM (81.9%), and there was a statistically significant association between sex and fracture type. Automobile traffic accidents were the major cause for all types of fractures. Work accidents were the second leading cause for FULM and ULM types, while bicycle/E-bike accidents were the second leading cause for FUL types. There was no significant difference between the cause of injury and fracture types (*p* = 0.339).

**Table 4:** Characteristics of panfacial fracture patients in relation to fracture types

Variable	Panfacial fracture					*p* value
	Total	FULM (%)	FUL (%)	ULM (%)	FUM (%)	
No. of patients	227	60	39	127	1	
Mean age	36.2 ± 14.3	37.6 ± 12.7	39.8 ± 13.9	34.5 ± 14.9	24	0.128
*Sex*						0.018*
Male	198	59 (98.3)	34 (87.2)	104 (81.9)	1 (100.0)	
Female	29	1 (1.7)	5 (12.8)	23 (18.1)	0 (0.0)	
*Injury cause*						0.339
Fall	31	5 (8.3)	2 (5.1)	24 (18.9)	0 (0.0)	
IPV	3	0 (0.0)	1 (2.6)	2 (1.6)	0 (0.0)	
AM acc	85	27 (45.0)	13 (33.3)	45 (35.4)	0 (0.0)	
B/E-B acc	44	11 (18.3)	11 (28.2)	21 (16.5)	1 (100.0)	
SO	17	4 (6.7)	5 (12.8)	8 (6.3)	0 (0.0)	
Work acc	47	13 (21.7)	7 (17.9)	27 (21.3)	0 (0.0)	
*Concomitant injuries*						0.014*
None	53	9 (15.0)	15 (38.5)	29 (22.8)	0 (0.0)	
One	85	16 (26.7)	15 (38.5)	54 (42.5)	0 (0.0)	
Two	53	20 (33.3)	6 (15.4)	26 (20.5)	1 (100.0)	
Three or more	36	15 (25.0)	3 (7.7)	18 (14.2)	0 (0.0)	
*Early surgical airway*						0.003*
None	179	38 (63.3)	36 (92.3)	104 (81.2)	1 (100.0)	
Yes	48	22 (36.7)	3 (7.7)	23 (18.1)	0 (0.0)	

IPV, Interpersonal violence; AM acc, Automobile accident; B/E-B acc, Bicycle/E-Bike accident; SO, Striking an object; *Significant difference

### Concomitant injuries

A total of 174 of the 227 patients had major concomitant injuries in other body parts. Traumatic craniocerebral injury was the most common concomitant injury, accounting for 46.3% of the patients in our cohort, followed by upper/lower limb injury (35.2%), thorax injury (34.8%), abdomen injury (9.7%), and cervical spine injury (8.8%). The FULM type had a high incidence of two or more categories of concomitant injuries, while the FUL type showed a higher correlation with no concomitant injuries (*p* = 0.014, Table 4). In addition, early surgical airway management was 36.7% in the FULM type, 18.1% in the ULM type, and 7.7% in the FUL type, showing a significant difference (*p* = 0.003, Table 4). Regarding the investigations for each category of concomitant injuries, as shown in Table 5 and Additional file 1: Table S2, thorax injuries and craniocerebral injuries were significantly correlated with the FULM type of fractures (*p* = 0.024 and *p* = 0.000, respectively), while upper/lower limb injuries were positively correlated with the ULM type and negatively correlated with the FUL type of fractures (*p* = 0.043 and *p* = 0.004, respectively).

**Table 5:** Statistical analysis of concomitant injuries and complications according to panfacial fracture types

Characteristic		FULM (n = 60)	FUL (n = 39)	ULM (n = 127)	FUM (n = 1)
*Major concomitant injuries*					
*p* value	Upper/lower limb injury (n = 79)	0.963	0.004^a^	0.043*	/
	Cervical spine injury (n = 20)	0.879	0.375	0.395	/
	Thorax injury (n = 79)	0.024*	0.092	0.539	/
	Cranio-Cerebral injury (n = 104)	0.000*	0.989	0.000^a^	/
	Abdomen injury (n = 22)	0.925	0.282	0.447	/
*Complications*					
*p* value	Pneumocranium (n = 34)	0.001*	0.120	0.000^a^	/
	Cerebral hematoma (n = 48)	0.000*	0.859	0.000^a^	/
	CSF leakage (n = 46)	0.010*	0.177	0.001^a^	/
	Hypoacusis (n = 12)	0.010*	0.406	0.106	/
	Diplopia (n = 33)	0.464	0.001*	0.091	/
	Hypopsia/blindness (n = 71)	0.024*	0.004*	0.000^a^	/
	Ocular movement limit (n = 49)	0.010*	0.050	0.000^a^	/
	Infraorbital nerve palsy (n = 104)	0.097	0.691	0.098	/
	Epiphora (n = 31)	0.220	0.393	0.092	/
	Anosmia (n = 26)	0.141	0.163	0.020^a^	/
	Ptosis (n = 35)	0.254	0.052	0.015^a^	/
	Traumatic facial palsy (n = 20)	0.151	0.033^a^	0.704	/
	Malocclusion (n = 189)	0.621	0.007^a^	0.015*	/
	Limited mouth opening (n = 152)	0.645	0.393	0.334	/

*Positive significant difference

^a^Negative significant difference

### Complications

Of all the patients, 97.8% complained of postinjury complications. The most common complication was malocclusion (83.7%), followed by limited mouth opening (67.4%), infraorbital nerve palsy (45.8%), and hypopsia/blindness (31.7%) (Additional file 1: Table S2). On statistical evaluation, complications showed a statistically significant correlation with fracture patterns. As shown in Table 5, FULM type showed a significantly positive correlation with pneumocranium (*p* = 0.001), cerebral hematoma (*p* = 0.000), CSF leakage (*p* = 0.010), hypoacusis (*p* = 0.010), hypopsia/blindness (*p* = 0.024), and ocular movement limit (*p* = 0.010); FUL type showed a significantly positive correlation with diplopia (*p* = 0.001) and hypopsia/blindness (*p* = 0.004) and a negative correlation with traumatic facial palsy (*p* = 0.033) and malocclusion (*p* = 0.007), while ULM types showed a significantly positive correlation only with malocclusion (*p* = 0.015) and a negative correlation with other complications, such as ptosis (*p* = 0.015) and anosmia (*p* = 0.020).

### FISS score

The mean FISS score assigned was 11.7 ± 4.5, and it was significantly higher in male patients (*p* = 0.007, Table 6). There was a significant correlation between fracture patterns and FISS scores (*p* = 0.000, Table 6). FULM type was related to the obviously highest FISS scores (14.7 ± 4.6), followed by FUL (11.4 ± 3.3) and ULM (10.3 ± 4.1). There was no significant difference between the cause of injuries and FISS scores (*p* = 0.559, Table 6). In addition, a significant difference was also found between FISS scores and concomitant injuries or complications. As shown in Table 7, statistically higher FISS scores were demonstrated in patients with major concomitant injuries, such as thorax injuries (*p* = 0.000), craniocerebral injuries (*p* = 0.027), and abdomen injuries (*p* = 0.031), and complications, such as hypoacusis (*p* = 0.004), cerebral hematoma (*p* = 0.024), anosmia (*p* = 0.002), and early surgical airway management (*p* = 0.000). Moreover, we performed ROC curve analysis to determine the value of the FISS score for the prediction of major concomitant injuries in patients with panfacial trauma, and the optimal cutoff value for the FISS score was 10.5 (AUC = 0.672, 95% CI 0.600–0.744, Additional file 1: Figure S1). We then classified the PF patients into a high FISS group (FISS ≥ 11, n = 124) and a low FISS group (FISS < 11, n = 103). Consistent with Table 7, the high FISS group showed a significant correlation with thorax injuries, craniocerebral injuries, early airway management, and the majority of complications (Additional file 1: Table S3).

**Table 6:** Characteristics panfacial fracture patients in relation to FISS score

Variable	FISS score (SD)	*p* value
*Sex*		0.007*
Male	12.0 (4.5)	
Female	9.6 (3.6)	
*Fracture type*		0.000*
FULM	14.7 (4.6)	
FUL	11.4 (3.3)	
ULM	10.3 (4.1)	
FUM	12 (/)	
*Injury cause*		0.559
Fall	11.5 (5.0)	
Interpersonal violence	8.3 (5.0)	
Automobile accident	11.4 (4.2)	
Bicycle/E-bike accident	11.8 (4.4)	
Striking an object	11.6 (3.8)	
Work accident	12.6 (4.8)	
*Concomitant injuries*		0.001*
None	10.4 (4.5)	
One	10.9 (4.3)	
Tow	13.0 (4.2)	
Three or more	13.4 (4.4)	

^*^Significant difference

**Table 7:** Statistical analysis of concomitant injuries and complications in relation to FISS score

Characteristics	FISS score (SD)		*p* value
	Yes	None	
*Major concomitant injuries*			
Upper/lower limb injury	11.9 (4.6)	11.6 (4.5)	0.55
Cervical spine injury	12.9 (3.8)	11.6 (4.5)	0.224
Thorax injury	13.1 (4.7)	10.9 (4.2)	0.000*
Cranio-cerebral injury	12.4 (4.2)	11.1 (4.7)	0.027*
Abdomen injury	13.6 (4.1)	11.5 (4.5)	0.031*
*Complications*			
Early surgical airway	14.67 (4.53)	10.88 (4.14)	0.000*
Pneumocranium	12.9 (3.7)	11.5 (4.6)	0.091
Cerebral hematoma	13.0 (3.8)	11.3 (4.6)	0.024*
CSF leakage	12.6 (4.4)	11.4 (4.5)	0.118
Hypoacusis	15.3 (5.5)	11.5 (4.4)	0.004*
Diplopia	13.1 (4.4)	11.4 (4.5)	0.056
Hypopsia/blindness	13.4 (4.8)	10.9 (4.1)	0.000*
Ocular movement limit	13.1 (4.6)	11.3 (4.4)	0.013*
Infraorbital nerve palsy	12.5 (4.6)	10.9 (4.3)	0.007*
Epiphora	14.4 (5.3)	11.3 (4.2)	0.000*
Anosmia	14.3 (4.1)	11.3 (4.4)	0.002*
Ptosis	12.0 (4.3)	11.6 (4.5)	0.621
Traumatic facial palsy	12.5 (4.7)	11.6 (4.5)	0.395
Malocclusion	11.9 (4.6)	10.6 (3.9)	0.126
Limited mouth opening	11.9 (4.7)	11.3 (4.0)	0.321

^*^Significant difference

**Fig. 5:**
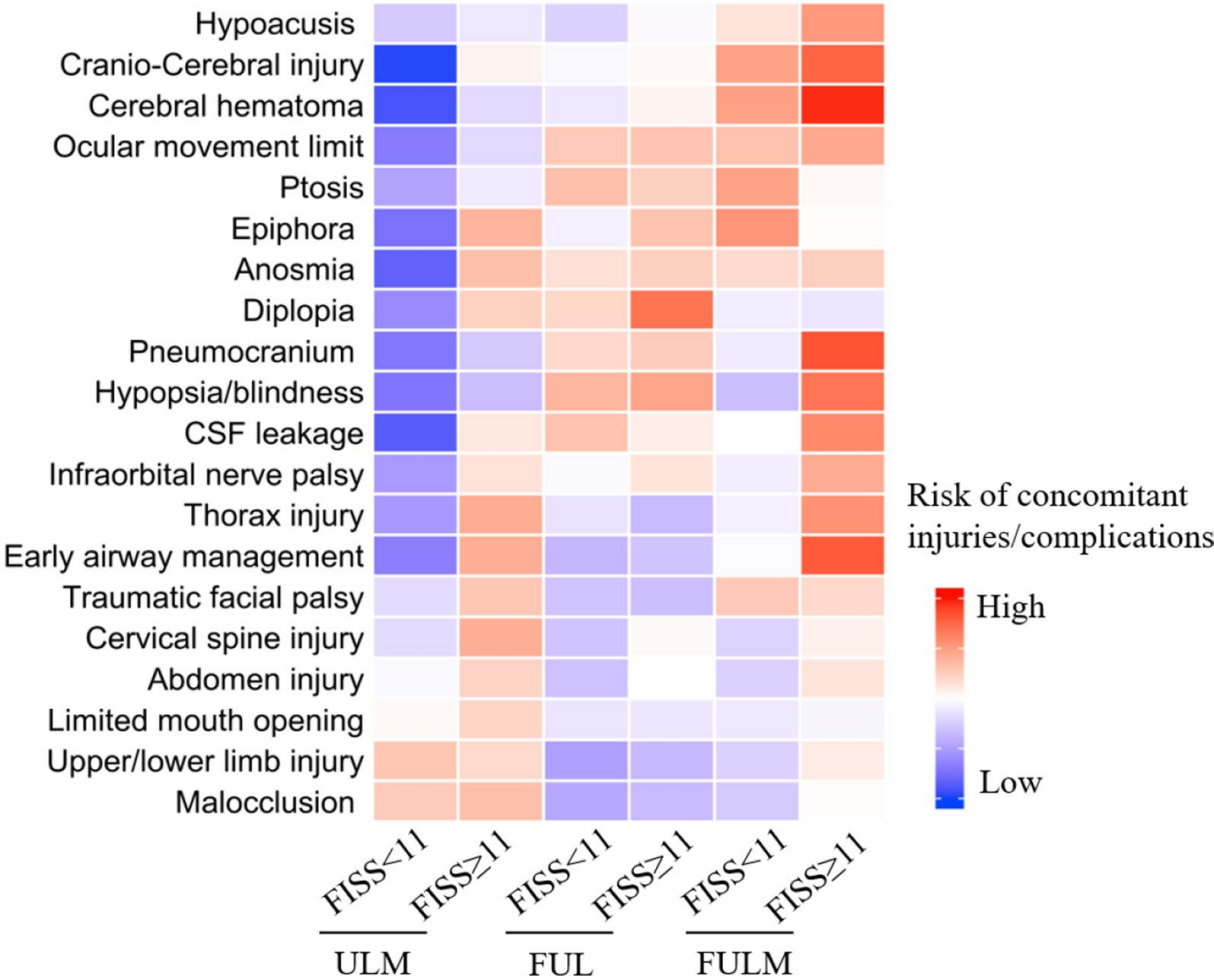
Visualization of the risk of concomitant injuries and complications with panfacial fracture types

To present the risks of concomitant injuries and complications of panfacial trauma more accurately and intuitively, we developed a modified model that combined the assessment of anatomic PF categories and the FISS. Patients diagnosed with PFs were defined into 6 groups, including FULM&FISS ≥ 11 group, FULM&FISS < 11 group, FUL&FISS ≥ 11 group, FUL&FISS < 11 group, ULM&FISS ≥ 11 group, ULM&FISS < 11 group, except the FUM type, which accounted for only one patient. Statistically, more detailed correlation between the panfacial fractures and concomitant injuries or complications were presented compared with the anatomic PF categories or FISS scores alone (Table 8, Additional file 1: Table S4-S5). Interestingly, the heat map could provide a good visualization of the risk of concomitant injuries and complications among each new group (Fig. 5).

**Table 8:** Statistical analysis of concomitant injuries and complications according to modified panfacial fracture patterns

Characteristics		FULM FISS ≥ 11	FULM FISS < 11	FUL FISS ≥ 11	FUL FISS < 11	ULM FISS ≥ 11	ULM FISS < 11
*Major concomitant injuries*							
*p* value	Upper/lower limb injury	0.644	0.488	0.164	0.048^a^	0.377	0.177
	Cervical spine injury	0.737	0.390	1.000	0.347	0.057	0.423
	Thorax injury	0.010*	1.000	0.177	0.514	0.050	0.016^a^
	Cranio-cerebral injury	0.000*	0.062	0.895	0.872	0.764	0.000^a^
	Abdomen injury	0.532	0.608	1.000	0.301	0.295	0.898
*Complications*							
*p* value	Early surgical airway	0.000*	1.000	0.276	0.165	0.053	0.002^a^
	Pneumocranium	0.000*	1.000	0.234	0.580	0.217	0.002^a^
	Cerebral hematoma	0.000*	0.025*	0.795	0.818	0.393	0.000^a^
	CSF leakage	0.006*	1.000	0.671	0.151	0.566	0.000^a^
	Hypoacusis	0.016*	1.000	1.000	0.620	0.861	0.356
	Diplopia	0.566	1.000	0.001*	0.538	0.274	0.010^a^
	Hypopsia/blindness	0.002*	0.245	0.033*	0.082	0.127	0.001^a^
	Ocular movement limit	0.043	0.292	0.170	0.207	0.393	0.002^a^
	Infraorbital nerve palsy	0.050	0.958	0.526	0.903	0.490	0.018^a^
	Epiphora	0.936	0.044*	0.155	1.000	0.073	0.001^a^
	Anosmia	0.255	0.719	0.431	0.735	0.131	0.000^a^
	Ptosis	0.898	0.028*	0.264	0.130	0.656	0.030^a^
	Traumatic facial palsy	0.370	0.480	0.275	0.347	0.178	0.423
	Malocclusion	0.948	0.446	0.110	0.041^a^	0.137	0.218
	Limited mouth opening	0.812	0.868	0.573	0.553	0.320	0.892

*Positive significant difference

^a^Negative significant difference

## Discussion

The definition of PFs is still controversial. Some studies deemed that exact PF must simultaneously involve the upper, middle, and lower thirds of the face [17, 18]. Other studies often considered that fractures involving two out of the three thirds of the face are sufficient to be classified as PF [19, 20]. Recently, some researchers defined the facial skeleton into four segments, including the frontal, upper, and lower parts of the midfacial and mandibular subunits, and proposed that facial fractures involving at least three out of four subunits can be defined as PF [1, 2]. This is also generally accepted. In the present study, we defined PFs as involving at least three out of four subunits of the face; accordingly, of all 227 patients reviewed, three major patterns were defined: FULM (n = 60), FUL (n = 39), ULM (n = 127), and only one patient classified as FUM. All patients suffered from fractures in the upper midface. This result was in line with a study of 99 PF patients performed by Jang et al. [1].

The most common fracture sites in our study were the maxillary sinus wall, followed by the mandible, zygomatic arch, nasal bone, orbital floor, zygomaticomaxillary complex, and Naso-orbito-ethmoid complex. As described by Park et al. [6], the most common fracture sites in their study of PFs were mandible, frontozygomaticomaxillary complex, and nasal bone. Dalena et al. [5] also proposed that the most common fracture sites of PFs were the orbital, front sinus, nasal bone and mandible. These data suggested that mandibular fractures and complex fractures of the upper midface were more likely to occur in panfacial injuries. Additionally, the type of distribution of mandibular fractures in PFs was described in the present study. The mandibular symphysis/parasymphysis, body, and condyle were the most common sites suffering panfacial injuries, which was in line with the study by Yang et al. [21]. We further developed a facial skeleton anatomic heat map estimating the level of fracture risks in panfacial injury patients. This heat map was proposed for the first time and provided a clear visualization that fractures in the upper midface, such as the zygomatic arch, nasal bone, orbital floor, zygomaticomaxillary complex, naso-orbito-ethmoid complex, or in the mandibular front and collum, were most likely to occur in panfacial injury patients.

Facial fractures are more prevalent in males, and there was a tendency in the present study (male/female = 6.8/1). In terms of the PF patterns, the FULM type was significantly more dominant in males than the FUL and ULM types. The most common etiology for all types of fractures was automobile traffic accidents, which was consistent with other studies. Work accidents were the second most common cause of FULM and ULM types, and bicycle/E-bike accidents were the second most common cause of FUL types. In addition, falls were another frequent cause, especially for the ULM type. Road traffic accidents such as automobile accidents and bicycle/E-Bike accidents are both correlated with injuries at high velocity and impact; accordingly, efforts should be made to increase traffic safety to reduce the incidence of these injuries.

According to the multicenter study conducted by Brucoli et al., the most frequent cause of maxillofacial injury was fall, while zygomatic fractures were the most frequently encountered injuries. Falls from a height were associated with a low FISS value with no associated concomitant injuries, while concomitant injuries were seen in 27.3% of patients. The study emphasized the frequency of involvement of females, and the high frequency of zygomatic fractures [22–24]. An absence of specific and predefined indications to treatment was associated with comorbidities. Elderly patients require specific attention and multidisciplinary collaboration in the diagnosis and sequencing of trauma treatment. A prudent attitude may be kept in selected cases, especially when severe comorbidities are associated and function is not impaired [25, 26].

PFs are frequently associated with various life-threatening concomitant injuries and complications, the causes of which are usually large external forces [3–5]. Hwang et al. [27] reported that neurological disorders and ophthalmic complications were the most common complications in patients with PFs. In the present study, 97.8% of the patients suffered concomitant injuries or postinjury complications, with five departments found to be mostly involved in managing these problems: neurosurgery, orthopedic surgery, ophthalmology, otolaryngology, and thoracic surgery. Based on fracture patterns, the patients suffering from FULM-type fractures had a significantly higher proportion of concomitant injuries and complications. The FULM type showed a significant correlation with thorax injuries, craniocerebral injuries, and complications, including pneumocranium, cerebral hematoma, CSF leakage, hypoacusis, hypopsia/blindness, and ocular movement limit. While the FUL type showed a significant correlation with diplopia and hypopsia/blindness, the ULM type showed a significant correlation with limb injuries and malocclusion. Additionally, early airway management is another major concern in the treatment of panfacial injuries. Over one-fifth of our patients required a surgical airway upon arrival or prior to arrival at the trauma bay. In terms of fracture patterns, early surgical airway management was most frequently performed in FULM- and ULM-type fractures. This suggests that surgeons in charge of panfacial injury patients must have a high index of suspicion for these life-threatening injuries. While the probability of concomitant injuries and complications correlated with each type of PF pattern were different, classifying patients first will therefore help the treating clinicians cooperate rapidly and closely with the relevant departments.

Another finding in the present study was that the FISS can be applied to evaluate the risk of concomitant injuries and complications of panfacial injuries. At present, a number of facial injury severity systems have been proposed, such as the Craniofacial Disruption Score (CDS) [28], Maxillofacial Injury Severity Scores (MFISS) [29], Facial Fracture Severity Score (FFSS) [30], FISS [12], and the ZS model [10]. A series of studies have been performed regarding the evaluation or comparison of facial fracture patients with various types of injury scales. The FISS has been considered to be the best available communication tool for multidisciplinary teams. Researchers have demonstrated that a higher FISS score indicates a strong correlation with specialist surgery, high treatment costs, and long hospitalization [13–16]. Aita et al. [13] showed that patients with FISS > 5 presented 18 times the chance of needing surgical intervention in the OR and a greater possibility of hospitalization longer than 3 days. In terms of panfacial injuries, there are still no studies performed with regard to the correlation between FISS and concomitant injuries and complications. Based on the present study, a significant association was found between the FISS scores and PF patterns, concomitant injuries, and complications, and a FISS value of 11 or more was the appropriate cutoff for the prediction. The results revealed that PFs with FISS ≥ 11 were significantly correlated with multiple concomitant injuries and complications, requiring involvement of other specialties. This finding is also of great value to prompt communication within multidisciplinary teams.

In the present study, we revealed that both the anatomic PF categories and FISS were significantly correlated with several concomitant injuries and complications. The combined assessment of these two variables was then performed in an attempt to further propose a better prediction of concomitant injuries and complications for panfacial injury patients. As expected, our modified model provided a more accurate correlation between panfacial fractures and concomitant injuries or complications. Additionally, the heat map presented a good positive and negative predictive visualization of concomitant injuries and complications. Canzi et al. [31, 32] developed the comprehensive facial injury (CFI) score, which originated from the FISS system, and classified facial fractures into 6 clusters according to the range of CFI scores, which was significantly correlated with overall surgical time and length of hospitalization. In our study, this was the first study to classify PF patients based on the combination of anatomic PF categories and the FISS system, and this model has demonstrated potential predictive value.

## Conclusions

In the present study, we investigated various factors associated with PFs, including patient demographics, causes of injury, location of fractures, major concomitant injuries, and postinjury complications. The most common fractured bones of PFs were the maxillary sinus wall, mandible, zygomatic arch, lateral orbital wall, and nasal bone. Traumatic craniocerebral injury, limb injury, and thorax injury were the most common concomitant injuries. There was a significant association between PF patterns and sex, concomitant injuries, early surgical airway management, and complications. Different PF patterns were significantly correlated with different types of concomitant injuries and complications. The FISS score also showed a significant difference with PF patterns, sex, concomitant injuries and complications. A FISS value of 11 or more is the appropriate cutoff for the prediction. We further proposed that the combination of PF categories and FISS can provide a better positive and negative prediction of concomitant injuries and complications for PF patients. It is suggested that this combined model could be a potential tool for the classification of PFs and therefore for the integration of multidisciplinary teams. Classifying PFs should be the first step toward systemic treatment.

## Supplementary Information

**Additional file 1.** Supplementary statistical analysis.

## Data Availability

The datasets used and/or analyzed during the current study are available from the corresponding author on reasonable request.

## Abbreviations

PF: Panfacial fracture
FISS: Facial Injury Severity Scale
F: Frontal area
U: Upper midface area
L: Lower midface area
M: Mandible area
