# Supplementary material for: Classifying and standardizing panfacial trauma according to anatomic categories and Facial Injury Severity Scale: a 10-year retrospective study
Source: BMC Oral Health. 2021 Nov 1;21:557. doi: 10.1186/s12903-021-01900-w (PMC8559398; doi:10.1186/s12903-021-01900-w)
Supplement: Supplementary file 1 — Additional file 1. Supplementary statistical analysis. [file 12903_2021_1900_MOESM1_ESM.docx]

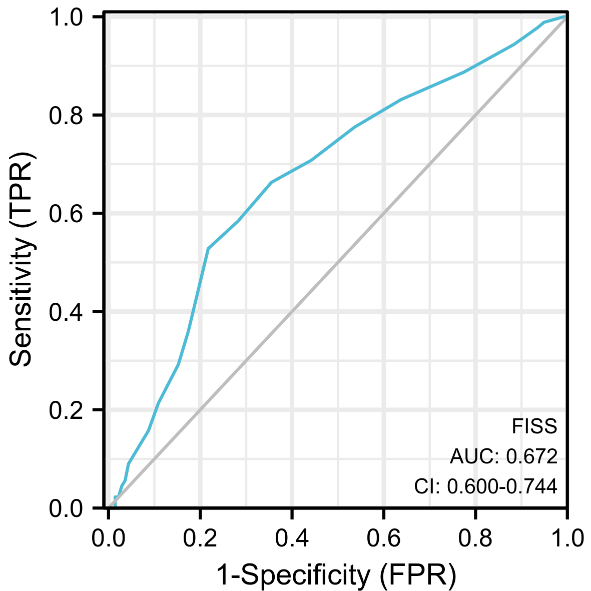


**Fig. S1.** Characteristics of the ROC curve analysis

**Table S1.** Age and gender of the patients

| Age (y) | Cases (%) | Gender (%) | |
| --- | --- | --- | --- |
|  |  | Male | Female |
| 0-18 | 19 (8.4) | 13 | 6 |
| 19-29 | 64 (28.2) | 54 | 10 |
| 30-39 | 50 (22.0) | 45 | 5 |
| 40-49 | 46 (20.3) | 44 | 2 |
| 50-59 | 39 (17.2) | 35 | 4 |
| 60-79 | 9 (3.9) | 7 | 2 |
| Total | 227 (100) | 198 (87.2) | 29 (12.8) |

**Table S2.** Statistical analysis of concomitant injuries and complications according to panfacial fracture types

| Characteristic | Total (n=227) | FULM (n=60) | FUL (n=39) | ULM (n=127) | FUM (n=1) | *p*-value |
| --- | --- | --- | --- | --- | --- | --- |
| Major concomitant injuries |  |  |  |  |  |  |
| Upper/lower limb injury | 80 | 21 | 6 | 52 | 1 | 0.016**^*^** |
| Cervical spine injury | 20 | 5 | 2 | 13 | 0 | 0.779 |
| Thorax injury | 79 | 28 | 9 | 42 | 0 | 0.079 |
| Cranio-Cerebral injury | 105 | 42 | 18 | 44 | 1 | 0.000**^*^** |
| Abdomen injury | 22 | 6 | 2 | 14 | 0 | 0.729 |
| Complications |  |  |  |  |  |  |
| Pneumocranium | 34 | 17 | 9 | 8 | 0 | 0.000**^*^** |
| Cerebral hematoma | 49 | 27 | 8 | 13 | 1 | 0.000**^*^** |
| CSF leakage | 46 | 19 | 11 | 16 | 0 | 0.011* |
| Hypoacusis | 12 | 7 | 1 | 4 | 0 | 0.083 |
| Diplopia | 33 | 7 | 12 | 14 | 0 | 0.018**^*^** |
| Hypopsia/blindness | 72 | 26 | 20 | 25 | 1 | 0.000**^*^** |
| Ocular movement limit | 49 | 20 | 13 | 16 | 0 | 0.002**^*^** |
| Infraorbital nerve palsy | 104 | 33 | 19 | 52 | 0 | 0.238 |
| Epiphora | 31 | 11 | 7 | 13 | 0 | 0.370 |
| Anosmia | 26 | 10 | 7 | 9 | 0 | 0.125 |
| Ptosis | 35 | 12 | 10 | 13 | 0 | 0.076 |
| Traumatic facial palsy | 20 | 8 | 0 | 12 | 0 | 0.141 |
| Malocclusion | 190 | 49 | 27 | 113 | 1 | 0.030**^*^** |
| Limited mouth opening | 153 | 39 | 24 | 89 | 1 | 0.645 |

^*^Significant difference

**Table S3.** Statistical analysis of concomitant injuries and complications according to FISS

|  | FISS≥11 (n=124) | FISS<11 (n=103) | *p*-value |
| --- | --- | --- | --- |
| Major concomitant injuries |  |  |  |
| Upper/lower limb injury (n=80) | 45 | 35 | 0.717 |
| Cervical spine injury (n=20) | 15 | 5 | 0.055 |
| Thorax injury (n=79) | 53 | 26 | 0.006**^*^** |
| Cranio-Cerebral injury (n=105) | 70 | 35 | 0.001**^*^** |
| Abdomen injury (n=22) | 15 | 7 | 0.179 |
| Complications |  |  |  |
| Early airway management (n=48) | 38 | 10 | 0.000**^*^** |
| Pneumocranium (n=34) | 26 | 8 | 0.006**^*^** |
| Cerebral hematoma(n=49) | 37 | 12 | 0.001**^*^** |
| CSF leakage(n=46) | 34 | 12 | 0.003**^*^** |
| Hypoacusis (n=12) | 9 | 3 | 0.233 |
| Diplopia (n=33) | 24 | 9 | 0.024**^*^** |
| Hypopsia/blindness (n=72) | 49 | 23 | 0.006**^*^** |
| Ocular movement limit (n=49) | 32 | 17 | 0.09 |
| Infraorbital nerve palsy (n=104) | 66 | 38 | 0.014**^*^** |
| Epiphora (n=31) | 23 | 8 | 0.019**^*^** |
| Anosmia (n=26) | 21 | 5 | 0.004**^*^** |
| Ptosis (n=35) | 20 | 15 | 0.745 |
| Traumatic facial palsy (n=20) | 13 | 7 | 0.329 |
| Malocclusion (n=190) | 105 | 85 | 0.662 |
| Limited mouth opening (n=153) | 85 | 68 | 0.686 |

^*^Significant difference

**Table S4.** Statistical analysis of concomitant injuries and complications according to new panfacial fracture patterns

| Characteristics | FULM  FISS≥11 | FULM  FISS<11 | FUL  FISS≥11 | FUL  FISS<11 | ULM  FISS≥11 | ULM  FISS<11 | p-value |
| --- | --- | --- | --- | --- | --- | --- | --- |
| No. of patients | 50 | 10 | 21 | 18 | 52 | 75 |  |
| Major concomitant injuries |  |  |  |  |  |  |  |
| Upper/lower limb injury (n=80) | 19 | 2 | 4 | 2 | 21 | 31 | 0.074 |
| Cervical spine injury (n=20) | 5 | 0 | 2 | 0 | 8 | 5 | 0.305 |
| Thorax injury (n=79) | 25 | 3 | 4 | 5 | 24 | 18 | 0.012**^*^** |
| Cranio-Cerebral injury (n=105) | 34 | 8 | 10 | 8 | 25 | 19 | 0.000**^*^** |
| Abdomen injury (n=22) | 6 | 0 | 2 | 0 | 7 | 7 | 0.528 |
| Complications |  |  |  |  |  |  |  |
| Early airway management (n=48) | 20 | 2 | 2 | 1 | 16 | 7 | 0.000**^*^** |
| Pneumocranium (n=34) | 16 | 1 | 5 | 4 | 5 | 3 | 0.001**^*^** |
| Cerebral hematoma (n=49) | 22 | 5 | 5 | 3 | 9 | 4 | 0.000**^*^** |
| CSF leakage (n=46) | 17 | 2 | 5 | 6 | 12 | 4 | 0.002**^*^** |
| Hypoacusis (n=12) | 6 | 1 | 1 | 0 | 2 | 2 | 0.208 |
| Diplopia (n=33) | 6 | 1 | 8 | 4 | 10 | 4 | 0.005**^*^** |
| Hypopsia/blindness (n=72) | 25 | 1 | 11 | 9 | 12 | 13 | 0.000**^*^** |
| Ocular movement limit (n=49) | 16 | 4 | 7 | 6 | 9 | 7 | 0.008**^*^** |
| Infraorbital nerve palsy (n=104) | 29 | 4 | 11 | 8 | 26 | 26 | 0.179 |
| Epiphora (n=31) | 7 | 4 | 5 | 2 | 11 | 2 | 0.003**^*^** |
| Anosmia (n=26) | 8 | 2 | 4 | 3 | 9 | 0 | 0.011**^*^** |
| Ptosis (n=35) | 8 | 4 | 5 | 5 | 7 | 6 | 0.048**^*^** |
| Traumatic facial palsy (n=20) | 6 | 2 | 0 | 0 | 7 | 5 | 0.170 |
| Malocclusion (n=190) | 42 | 7 | 15 | 12 | 47 | 66 | 0.070 |
| Limited mouth opening (n=153) | 33 | 6 | 13 | 11 | 38 | 51 | 0.892 |

^*^Significant difference

**Table S5.** Phi relation value of concomitant injuries and complications according to new panfacial fracture patterns

| Characteristics | | FULM  FISS≥11 | FULM  FISS<11 | FUL  FISS≥11 | FUL  FISS<11 | ULM  FISS≥11 | ULM  FISS<11 |
| --- | --- | --- | --- | --- | --- | --- | --- |
| Major concomitant injuries | |  |  |  |  |  |  |
| *Phi relation* | Upper/lower limb injury | 0.031 | -0.068 | -0.108 | -0.148 | 0.059 | 0.09 |
|  | Cervical spine injury | 0.022 | -0.067 | 0.008 | -0.091 | 0.126 | -0.053 |
|  | Thorax injury | 0.17 | -0.022 | -0.106 | -0.043 | 0.13 | -0.159 |
|  | Cranio-Cerebral injury | 0.232 | 0.145 | 0.009 | -0.011 | 0.02 | -0.295 |
|  | Abdomen injury | 0.041 | -0.07 | -0.002 | -0.096 | 0.069 | -0.009 |
| Complications | |  |  |  |  |  |  |
| *Phi relation* | Early surgical airway | 0.245 | -0.006 | -0.091 | -0.112 | 0.128 | -0.203 |
|  | Pneumocranium | 0.254 | -0.03 | 0.079 | 0.06 | -0.082 | -0.216 |
|  | Cerebral hematoma | 0.29 | 0.148 | 0.017 | -0.035 | -0.057 | -0.277 |
|  | CSF leakage | 0.182 | -0.001 | 0.028 | 0.095 | 0.038 | -0.261 |
|  | Hypoacusis | 0.159 | 0.045 | -0.007 | -0.069 | -0.035 | -0.082 |
|  | Diplopia | -0.038 | -0.028 | 0.213 | 0.064 | 0.073 | -0.183 |
|  | Hypopsia/blindness | 0.209 | -0.1 | 0.142 | 0.115 | -0.101 | -0.217 |
|  | Ocular movement limit | 0.135 | 0.096 | 0.091 | 0.084 | -0.057 | -0.209 |
|  | Infraorbital nerve palsy | 0.13 | -0.025 | 0.042 | -0.008 | 0.046 | -0.157 |
|  | Epiphora | 0.005 | 0.165 | 0.094 | -0.022 | 0.119 | -0.225 |
|  | Anosmia | 0.076 | 0.058 | 0.076 | 0.048 | 0.1 | -0.253 |
|  | Ptosis | 0.009 | 0.146 | 0.074 | 0.1 | -0.03 | -0.144 |
|  | Traumatic facial palsy | 0.06 | 0.085 | -0.099 | -0.091 | 0.089 | -0.053 |
|  | Malocclusion | 0.004 | -0.08 | -0.106 | -0.135 | 0.099 | 0.082 |
|  | Limited mouth opening | -0.016 | -0.034 | -0.037 | -0.039 | 0.066 | 0.009 |
